# Mobile health apps for older adults: real-world evidence on engagement and medication adherence

**DOI:** 10.3389/fdgth.2026.1716880

**Published:** 2026-04-17

**Authors:** Elyssa Wiecek, Scott Taylor, Hugo Rourke, Fiona Hammond, Noelia Amador-Fernandez

**Affiliations:** 1 Perx Health, New York, NY, United States; 2 Graduate School of Health, University of Technology Sydney, Sydney, NSW, Australia

**Keywords:** aged, medication adherence, mHealth, mobile applications, patient compliance, telemedicine

## Abstract

**Introduction:**

A rapidly aging global population is placing increasing strain on healthcare systems. Digital health (mHealth) applications may support older adults in managing chronic conditions and adhering to medication, yet this population is often underrepresented in research. This study aimed to investigate engagement, retention, and adherence among adults aged ≥65 years using the Perx Health mobile app.

**Methods:**

We conducted a retrospective observational analysis using real-world data from Perx users in Australia and the United States. Medication adherence, app engagement, and retention patterns were analyzed among participants aged 65 years and older.

**Results:**

A total of 250 participants were included (mean age 70.1 ± 4.7 years; 61.8% female; 61.6% Australia-based). Median medication adherence was 95.0% (IQR 85.3–98.3). Participants were retained in the app for a median of 595.5 days (IQR 206.3–1,182.8). Older adults demonstrated higher engagement with gamified and social features than the overall Perx user population, with 62.5% engaging with leaderboards and 26.5% using streak tracking.

**Discussion/conclusion:**

Older adults demonstrated high engagement, adherence, and retention with the Perx app. These findings suggest that mHealth solutions incorporating social and gamified elements can effectively support self-management in this population. The results provide a foundation for future research on app design features that optimize uptake and sustained use among older adults.

## Introduction

The global population is aging rapidly with the number and proportion of people aged 60 expanding quickly in nations worldwide. This demographic shift is one of the most significant global trends of the 21st century (1). By 2030, one in six people worldwide will be aged 60 or older, and by 2050, the global population of people aged 60 and above will have more than doubled since 2015, reaching 2.1 billion people (1, 2). This demographic shift will have significant implications for the health and well-being of society as a whole. An aging population is often accompanied by an increased prevalence of multiple comorbidities, placing considerable strain on already overburdened healthcare systems (3). A key challenge arising from this complexity is poor medication adherence, which is common in older adults with multiple chronic conditions and is a major determinant of poor health outcomes (4).

At the same time, older adults are remaining independent longer than previous generations (5). However, healthcare systems and aged care services often fail to adequately involve them in the planning and implementation of their care as they age (6, 7). This lack of engagement and empowerment stands in contrast to older adults' strong preference for maintaining independence in later life (8). Ensuring that they are active partners in their health management is therefore critical to supporting both their autonomy and their well-being.

One avenue with particular promise for the empowerment of older adults is the use of digital health tools. However, many mobile health applications are not explicitly designed with older adults in mind, often overlooking usability needs related to age-related changes in cognition and interaction (9–11). This may stem from assumptions that older adults are less interested in, or less likely to adopt, innovative technologies. In contrast, recent research demonstrates that older adults can engage effectively with digital health tools when usability and accessibility are appropriately considered (12–15).

Yet despite the growing evidence that older adults can and do use mobile health technologies, relatively little is known about how older adults engage with such tools in real-world contexts, or how this engagement translates into effective health management (16, 17). Understanding these engagement patterns is critical to inform future design and implementation. Our study addresses this gap by examining multi-country, real-world engagement and longitudinal retention over >12 months, including feature-level usage patterns, among older adult users of the Perx app.

The aim of this study was to use real-world data to investigate how adults aged 65 years and older engage with a mobile health (mHealth) application designed to support the management of chronic conditions and adherence to medication, including patterns of app use and retention. By examining patterns of engagement, we sought to better understand how older adults use and benefit from digital health tools.

## Materials and methods

### Study design

This study was a retrospective observational analysis using real-world data from the Perx Health mobile application in both the United States and Australia. Methods and reporting followed the STROBE guidelines for observational studies, and the RECORD extension, appropriate for studies using routinely collected digital health data (Supplementary Material: STROBE checklist).

### Intervention—the Perx mobile app program

The Perx mobile app is a digitally scaled health engagement platform designed to support members in following their treatment plans. Grounded in behavioral science, it incorporates games, rewards, and social recognition to provide a multifaceted motivation platform. Users enter their own treatment plans, including medications, exercise therapy, medical appointments, or other daily health tasks, and receive reminders based on the schedules they set.

Medication adherence is self-reported and verified using mobile Directly Observed Therapy (mDOT), whereby users upload a photo of themselves taking their medication. Educational content, such as short “fun facts” or program-specific information (e.g., insurance details), may also be presented as daily tasks. Users are motivated to complete tasks through lottery-style rewards, point accumulation with level progression, streaks for consecutive daily completions, and social features such as leaderboards and community forums.

### Participant recruitment and data source

Users were eligible to use the Perx app if invited by a program sponsor. Sponsors varied and included private health insurers, workers' compensation insurers, public health programs, private clinics, and patient advocacy groups. Invitations were sent via email or SMS (Short Message Service), with no clinical staff typically involved in enrollment. As access is only provided via program sponsors, users were unable to independently enroll in a public free version of the app. In some cases, users may retain access to a non-incentivized version following the conclusion of a sponsorship period, during which monetary rewards are no longer available yet core functionality remains unchanged.

User sign-up dates ranged from November 2016 to March 2025. Due to data retention policies, detailed adherence and engagement data were available from January 1, 2021 onward. Retention was calculated based on time since original sign-up date, including for users who enrolled prior to January 1, 2021 but remained active during the observation period. Adherence and engagement analyses were restricted to the period for which data were available.

Inclusion criteria for this analysis were: (1) a self-reported date of birth indicating age ≥65 years at sign-up, and (2) engagement with the app for at least 7 days. This requirement was applied to ensure the analysis captured meaningful interactions with the tool's features rather than “floor effects” or “noise” caused by immediate attrition during the initial onboarding phase. A 7-day period allows for a stabilization of usage patterns, distinguishing users who have successfully oriented themselves to the digital interface from those who discontinued use due to external factors or technical barriers unrelated to the study's objectives. A supplementary sensitivity analysis was conducted including all users with scheduled medication tasks regardless of engagement duration to assess the impact of the ≥7-day inclusion criterion on adherence estimates.

User data from the Perx database were used to evaluate adherence to medications and other health tasks, including physical therapy, clinical measurements, or custom tasks, as well as daily log-ins to measure retention. All data were fully de-identified prior to analysis.

Key demographic variables such as date of birth and clinical conditions were optional. Users missing these fields were excluded from analyses requiring them. Analyses of medication adherence included only users who completed at least one medication task. For adherence calculations, days with no recorded adherence during active app use were treated as 0% until a user was inactive for 3 consecutive days, per our adherence definition.

### Outcomes and data analysis

Medication adherence rates were defined as the number of doses taken over the number of doses scheduled during each user's active period and averaged per day. Days with no recorded adherence between active periods were counted as 0% until the user was no longer active (retained) after 3 days. This approach was chosen after testing alternative inactivity windows, which showed that results were robust to different tail lengths, with the 3-day tail closely approximating more complex historical calculation methods. Adherence was averaged per user over their active period and then aggregated across the cohort. Because adherence data are typically skewed with wide ranges, adherence rates were summarized using the median and interquartile range (IQR). Mean and standard deviation were also reported for completeness.

Retention was defined as the total number of days between the user's first login day and last day they completed any task (their own scheduled health task or a Perx program-prompted task).

Engagement with specific features was assessed using Amplitude digital analytics software, which captured navigation to feature screens (e.g., social forum, leaderboard, rewards tab). App engagement evaluated sessions per day per user, time spent in the app and completed tasks per day. A session begins when the app moves into the foreground and ends when the app goes into the background with no events fired for at least 5 min. All events sent within 5 min of each other are counted as part of the current session, and background processes do not constitute separate sessions. Feature usage was defined as the proportion of users who navigated to a given feature (e.g., leaderboard, rewards tab) during a 1-month period, averaged across the previous 12 months of data. For reporting, feature-specific usage rates were normalized by expressing them as percent differences relative to Perx's standard feature usage baselines, which comprised the broader Perx user population across all programs during the same observation period, thereby avoiding disclosure of absolute usage values.

Analyses were conducted in R (RStudio version 2) and Microsoft Excel 2025 (Microsoft Corporation). Continuous variables (adherence, retention) were summarized using mean, standard deviation, median, and interquartile range. Categorical variables (feature usage) were summarized as counts and percentages.

### Ethics statement and data security

This study was approved by the University of Technology Sydney Human Research Ethics Committee in August 2025 (HREC; approval number ETH25-10992). All Perx users provided active informed consent to the Terms of Use and Privacy Policy, which state that de-identified, aggregated data may be used by third parties for research and other purposes. No personal or confidential data were included in the database. For mobile directly observed therapy (mDOT) photos, the following safeguards were in place: photos were automatically de-identified upon upload, stored in a secure, access-controlled environment, and deleted according to Perx's data retention policies. Only de-identified, aggregated engagement and adherence metrics were included in the research database. Perx Health maintains SOC 2 Type II attestation via independent auditors, and applies HIPAA-aligned administrative, technical, and physical safeguards for protected health information.

## Results

### Participants

A total of 250 users were included in the analysis. The mean age was 70.1 years (SD = 4.7), with a range from 65 to 87, as seen in Table 1. Most participants were female (61.8%, *n* = 155) and were located in Australia (61.6%, *n* = 154). Users were enrolled across diverse sponsor programs, including health providers (36.8%, *n* = 92), private insurers (20.8%, *n* = 52), general health programs (20.4%, *n* = 51), workers' compensation (13.6%, *n* = 34), and public health programs (8.4%, *n* = 21). Among the 249 users who reported at least one clinical condition, most had multiple conditions, with 62 (24.9%) reporting one condition, 21 (8.4%) reporting two conditions, and 167 (67.0%) reporting three or more conditions, indicating a high prevalence of multi-morbidity in this cohort. Reported clinical conditions included heart and circulation conditions (33.6%, *n* = 84), other conditions such as allergies, obesity management, and chronic pain (29.6%, *n* = 74), and musculoskeletal and rheumatological conditions (22.0%, *n* = 55). On average, users had 9.5 scheduled medication tasks, corresponding to the number of distinct prescribed medications, with each task counted once regardless of how frequently the medication was taken (e.g., daily, weekly).

**Table 1:** Baseline characteristics of the study cohort.

Variable	*n* (%)
Age (mean ± SD) (years) (*N* = 250)	70.1 ± 4.7
Sex (*N* = 250)	
Female	155 (61.8%)
Male	95 (38.2%)
Location (*N* = 250)	
Australia	154 (61.6%)
US	96 (38.4%)
Clinical conditions	
Heart and circulation	84 (33.6%)
Other (e.g., allergies, obesity management, chronic pain)	74 (29.6%)
Musculoskeletal and rheumatological	55 (22.0%)
Endocrine	49 (19.6%)
Injury	36 (14.4%)
Lung and breathing	35 (14.0%)
Mental health	30 (12.0%)
Ophthalmological	23 (9.2%)
Digestive	18 (7.2%)
Cancer	16 (6.4%)
Neurological	13 (5.2%)
Kidney and bladder	13 (5.2%)
Skin conditions	11 (4.4%)
Transplant	6 (2.4%)
Addiction medicine	2 (0.8%)
Infectious diseases	2 (0.8%)
Chronic conditions	1 (0.4%)
Number of reported clinical conditions	
1 Condition	62 (24.9%)
2 Conditions	21 (8.4%)
3 Conditions	167 (67.0%)
Number that reported a condition	249 (100.0%)
Sponsor type (*N* = 250)	
Provider	92 (36.8%)
Private health insurer	52 (20.8%)
General health	51 (20.4%)
Workers’ compensation	34 (13.6%)
Public health	21 (8.4%)

### Medication adherence

Median medication adherence across the cohort was 95.0% (IQR = 85.3–98.3) and average adherence was 86.2% (SD = 20.4) (Table 2). When stratified by program sponsor type, median adherence was broadly similar across programs, ranging from 93.2% in the General Health program to 97.6% in Public Health programs, with mean adherence ranging from 77.5% to 91.8% (Table 3).

**Table 2:** Medication adherence and participant retention.

Outcome	Median (IQR)	Mean (SD)
Medication adherence (average rate)	95.0 (85.3–98.3)	86.2 (20.4)
Retention (days)	595.5 (206.3–1,182.8)	779.7 (736.2)

**Table 3:** Medication adherence and sponsor type.

Program type	Count (*N* = 250)	Adherence median (IQR)	Adherence mean (SD)
General health	92 (36.8%)	93.2 (67.7–98.2)	77.5 (29.4)
Private health insurer	52 (20.8%)	96.9 (86.0–99.0)	86.8 (21.5)
Provider	51 (20.4%)	93.6 (85.3–97.0)	88.5 (13.4)
Public health	34 (13.6%)	97.6 (92.5–99.2)	91.8 (17.9)
Workers’ compensation	21 (8.4%)	96.1 (87.8–99.0)	89.0 (16.6)

Inclusion of early disengaged users in a sensitivity analysis yielded similar average adherence estimates (84.2% vs. 86.2% in the primary cohort; Supplementary Table S1), suggesting findings were not materially driven by the engagement threshold.

### Retention

Participants were retained in the app for a mean of over 2 years, 779.7 days (SD = 736.2), with a median of over one and a half years, 595.5 days (IQR = 206.3–1,182.8). The maximum retention observed was 2,465 days (Table 2).

### App and feature engagement

Participants logged into the app on average 6.0 times per day. They spent on average 8 min and 35 s in the app daily, or 3 h and 23 min per month. They completed on average 4.9 tasks per day.

Feature usage varied across the cohort as seen in Table 4. Relative to Perx's standard baselines, the largest increases were observed for leaderboard (62.5%) and streak tracking (26.5%), reflecting strong engagement with social-comparative and gamified progress-tracking features. More modest increases were seen for the community forum (17.1%) and health history (3.1%), while usage of the rewards tab decreased by 12.8%.

**Table 4:** Feature engagement of older adults relative to Perx baselines.

Feature	Percent difference (%)	Interpretation category
Leaderboard	+62.5	Social–comparative/gamified
Streak tracking	+26.5	Progress-tracking/gamified
Community forum	+17.1	Social/community
Health history	+3.1	Informational/self-monitoring
Rewards tab	−12.8	Incentives/extrinsic

## Discussion

In this multi-country, real-world cohort, older adults using the Perx app were observed to have high levels of engagement, retention, and adherence to their health tasks including medication adherence. Their usage patterns indicated relatively greater access to the social and leaderboard features of the app and less use of the rewards feature compared to Perx's overall averages.

Medication adherence has previously been evaluated for the Perx app in both a retrospective real-world analysis and a randomized controlled trial (18, 19). The median adherence rate among older adults in this study (95.0%) was similar to those earlier findings, suggesting that engaged users of the Perx program tend to report high adherence. Importantly, adherence remained broadly consistent across program sponsor types, suggesting that high adherence was not confined to a single delivery or incentive context. A review of long-term medication regimens in older adults estimates adherence at around 50%, consistent with WHO findings that only about half of patients with chronic conditions adhere to prescribed medications (20, 21). While our study did not directly measure clinical outcomes, prior evidence suggests that high adherence is associated with improved outcomes and reduced healthcare costs (19, 22).

Retention among older adults in the Perx app was also notably high, with a median of 595.5 days. By comparison, historical analyses of the general Perx population have shown an average of 242 days, suggesting that older adults may be more likely to remain engaged for longer periods. These findings are consistent with previous research on digital health interventions. A large meta-analysis of over 100,000 participants across eight remote digital health studies reported median engagement of only 5.5 days (range 2–26 days), with older age (≥60 years) associated with an increase in engagement of four additional days (23). In contrast, our real-world evidence demonstrates that older adults retained in the Perx program for substantially longer, exceeding the averages reported in prior digital health literature.

The reasons why older adults sustain engagement with digital health programs remain understudied. Prior work suggests that older adults tend to be motivated more by intrinsic drivers, such as health improvements or social support, than by extrinsic rewards (24). For example, a study comparing younger and older adults using a balance-training exergame found that older adults were motivated primarily by perceived health benefits, while younger adults were motivated by in-game rewards. Our findings are consistent with this distinction: older adults were approximately 12.8% less likely than the general Perx population to access the rewards feature regularly, suggesting that financial incentives may be less important to them. However, as this was a real-world study, we did not collect detailed socioeconomic data on participants. Therefore, while financial incentives appeared less important in this sample, we cannot determine whether this pattern would hold across older adults with differing income or financial circumstances.

In contrast, older adults in our cohort engaged more frequently with social features of the Perx app than the overall population, being 17.1% more likely to engage with them. This interpretation aligns with prior research demonstrating that older adults are more responsive to social and health-related motivators. For example, in a study evaluating attitudes toward activity trackers, older adults reported being positively influenced by knowing others were also using the devices (25). Similarly, other research has shown that praise or social feedback can be especially motivating for older adults compared to younger participants (26). It is possible that older Perx users sought out similar social reinforcement through leaderboards and community features, complementing their high adherence to daily tasks.

### Study strengths and limitations

This study is among the first to evaluate real-world engagement with a mobile health program specifically among adults aged 65 years and older, a group that is often underrepresented in digital health research. Unlike many prior studies relying on short-term trials or self-report, we analyzed large-scale, routinely collected app data, allowing for objective measurement of adherence, retention, and feature use over extended periods. The inclusion of participants across diverse program types in both the United States and Australia further enhances the relevance of our findings.

This study also has some limitations. First, entering date of birth in the Perx app is optional, meaning some older users may have been missed in the study. Second, we were unable to establish baseline adherence rates prior to program use or to include a control group, given the retrospective design. Third, as the study spanned >12 months, routine software updates and intermittent technical issues may have affected usability for some participants, which could have influenced engagement, retention, and adherence over time.

Additionally, feature engagement analyses were limited to counts of users accessing each feature in a given time period. While these provide insight into behavioral patterns, motivations for engagement were inferred rather than directly measured. Accordingly, findings are descriptive and associational, and causal inferences regarding the impact of specific app features cannot be made. Due to ethics and privacy constraints, raw participant-level data cannot be made publicly available. While de-identified data may be shared upon reasonable request and subject to institutional data governance approvals, this restriction may limit independent reproducibility.

Finally, we acknowledge the inherent survival bias introduced by our 7-day inclusion criterion. In digital health research, there is no standardized consensus on the optimal threshold for defining an “active user,” and investigators frequently struggle to balance the inclusion of all participants with the need for high-quality engagement data. We excluded individuals who discontinued use within the first week because this initial onboarding period often reflects technical orientation or immediate attrition rather than the sustained engagement patterns our study sought to investigate. Furthermore, because our methodology includes a 3-day data “tail,” early-attrition users would be assigned multiple days of 0% adherence, creating a floor effect that disproportionately weights non-usage over actual behavioral interaction. While this approach allows for a clearer examination of how older adults use and benefit from the tool over months of use, it may lead to an overestimation of adherence and retention metrics relative to the total population of initial adopters. Accordingly, our adherence and retention estimates are conditional on ≥7 days of engagement and are most generalizable to older adults who become ongoing users, not all initiators.

### Future directions

These findings offer several important considerations for both future design and evaluation of mHealth strategies. The high observed median retention rate of 595.5 days is consistent with the possibility that when designed appropriately, digital health solutions can foster long-term engagement in older adults. Future studies around older adults' engagement with mHealth should leverage detailed usage patterns to understand and target user drop-off points. Furthermore, while a Randomized Controlled Trial (RCT) has been conducted with Perx Health to rigorously evaluate clinical outcomes, these designs should now be specifically utilized to observe vulnerable populations such as the greater than 65 year old demographic (19). This further research will be essential to solidify the evidence base for digital interventions targeting the aging global population.

## Conclusion

In summary, older adults in this selected user cohort demonstrated sustained engagement, adherence, and retention in the Perx app, with use far exceeding averages reported for digital health interventions more broadly. Their relatively higher use of social features suggests that digital programs aiming to support older adults may benefit from intrinsic and socially driven motivators, potentially even more so than programs designed for other cohorts. These findings should be interpreted in the context of the study's limitations, including the nature of the user group, but they highlight the potential of mHealth solutions to empower older adults in managing their health and provide a foundation for future research exploring the specific design elements that best support this growing population.

## Data Availability

The data analyzed in this study is subject to the following licenses/restrictions: due to ethics approval, we are unable to share the raw, unidentifiable data used in this analysis. Requests to access these datasets should be directed to Elyssa Wiecek, elyssa@perxhealth.com.
