# Supplementary material for: Mobile health apps for older adults: real-world evidence on engagement and medication adherence
Source: Front Digit Health. 2026 Apr 17;8:1716880. doi: 10.3389/fdgth.2026.1716880 (PMC13132811; doi:10.3389/fdgth.2026.1716880)
Supplement: Supplementary file 1 [file Supplementaryfile1.docx]

STROBE Statement—checklist of items that should be included in reports of observational studies

|  | Item No. | Recommendation | Page  No. | Relevant text from manuscript |
| --- | --- | --- | --- | --- |
| **Title and abstract** | 1 | (*a*) Indicate the study’s design with a commonly used term in the title or the abstract | Materials and methods – Study design | This study was a retrospective observational analysis using real-world data from the Perx Health mobile application in both the United States and Australia. Methods and reporting followed the STROBE guidelines for observational studies, and the RECORD extension, appropriate for studies using routinely collected digital health data. |
|  |  | (*b*) Provide in the abstract an informative and balanced summary of what was done and what was found | Abstract | We conducted a retrospective observational analysis using real-world data from Perx users in Australia and the United States.  Medication adherence, app engagement, and retention patterns were analyzed among participants aged 65 years and older. |
| Introduction | | | |  |
| Background/rationale | 2 | Explain the scientific background and rationale for the investigation being reported | Introduction | Yet despite the growing evidence that older adults can and do use mobile health technologies, relatively little is known about how older adults engage with such tools in real-world contexts, or how this engagement translates into effective health management. (16,17) Understanding these engagement patterns is critical to inform future design and implementation. Our study addresses this gap by examining multi-country, real-world engagement and longitudinal retention over >12 months, including feature-level usage patterns, among older adult users of the Perx app. |
| Objectives | 3 | State specific objectives, including any prespecified hypotheses | Introduction | The aim of this study was to use real-world data to investigate how adults aged 65 years and older engage with a mobile health (mHealth) application designed to support the management of chronic conditions and adherence to medication, including patterns of app use and retention. By examining patterns of engagement, we sought to better understand how older adults use and benefit from digital health tools. |
| Methods | | | |  |
| Study design | 4 | Present key elements of study design early in the paper | Materials and methods – Study design | This study was a retrospective observational analysis using real-world data from the Perx Health mobile application in both the United States and Australia. Methods and reporting followed the STROBE guidelines for observational studies, and the RECORD extension, appropriate for studies using routinely collected digital health data. |
| Setting | 5 | Describe the setting, locations, and relevant dates, including periods of recruitment, exposure, follow-up, and data collection | Materials and methods – Participant recruitment and data source | Users were eligible to use the Perx app if invited by a program sponsor. Sponsors varied and included private health insurers, workers’ compensation insurers, public health programs, private clinics, and patient advocacy groups. Invitations were sent via email or SMS (Short Message Service), with no clinical staff typically involved in enrollment.  User sign-up dates ranged from November 2016 to March 2025. Due to data retention policies, detailed adherence and engagement data were available from January 1, 2021 onward. Retention was calculated based on time since original sign-up date, including for users who enrolled prior to January 1, 2021 but remained active during the observation period. Adherence and engagement analyses were restricted to the period for which data were available. |
| Participants | 6 | (*a*) *Cohort study*—Give the eligibility criteria, and the sources and methods of selection of participants. Describe methods of follow-up  *Case-control study*—Give the eligibility criteria, and the sources and methods of case ascertainment and control selection. Give the rationale for the choice of cases and controls  *Cross-sectional study*—Give the eligibility criteria, and the sources and methods of selection of participants | n/a |  |
|  |  | (*b*) *Cohort study*—For matched studies, give matching criteria and number of exposed and unexposed  *Case-control study*—For matched studies, give matching criteria and the number of controls per case | n/a |  |
| Variables | 7 | Clearly define all outcomes, exposures, predictors, potential confounders, and effect modifiers. Give diagnostic criteria, if applicable | Materials and methods – Outcomes and data analysis | Medication adherence rates were defined as the number of doses taken over the number of doses scheduled during each user’s active period and averaged per day. Days with no recorded adherence between active periods were counted as 0% until the user was no longer active (retained) after three days. This approach was chosen after testing alternative inactivity windows, which showed that results were robust to different tail lengths, with the three-day tail closely approximating more complex historical calculation methods. Adherence was averaged per user over their active period and then aggregated across the cohort. Because adherence data are typically skewed with wide ranges, adherence rates were summarized using the median and interquartile range (IQR). Mean and standard deviation were also reported for completeness.  Retention was defined as the total number of days between the user’s first login day and last day they completed any task (their own scheduled health task or a Perx program-prompted task).  Engagement with specific features was assessed using Amplitude digital analytics software, which captured navigation to feature screens (e.g., social forum, leaderboard, rewards tab). App engagement evaluated sessions per day per user, time spent in the app and completed tasks per day. A session begins when the app moves into the foreground and ends when the app goes into the background with no events fired for at least five minutes. All events sent within five minutes of each other are counted as part of the current session, and background processes do not constitute separate sessions. Feature usage was defined as the proportion of users who navigated to a given feature (e.g., leaderboard, rewards tab) during a one-month period, averaged across the previous 12 months of data. For reporting, feature-specific usage rates were normalized by expressing them as percent differences relative to Perx’s standard feature usage baselines, thereby avoiding disclosure of absolute usage values.  Analyses were conducted in R (RStudio version 2) and Microsoft Excel 2025 (Microsoft Corporation). Continuous variables (adherence, retention) were summarized using mean, standard deviation, median, and interquartile range. Categorical variables (feature usage) were summarized as counts and percentages. |
| Data sources/ measurement | 8* | For each variable of interest, give sources of data and details of methods of assessment (measurement). Describe comparability of assessment methods if there is more than one group | Materials and methods |  |
| Bias | 9 | Describe any efforts to address potential sources of bias | Materials and methods & Discussion – Study strengths and limitations | This requirement was applied to ensure the analysis captured meaningful interactions with the tool's features rather than 'floor effects' or 'noise' caused by immediate attrition during the initial onboarding phase. A seven-day period allows for a stabilization of usage patterns, distinguishing users who have successfully oriented themselves to the digital interface from those who discontinued use due to external factors or technical barriers unrelated to the study’s objectives. |
| Study size | 10 | Explain how the study size was arrived at | Materials and methods – Participant recruitment and data source | Inclusion criteria for this analysis were: (1) a self-reported date of birth indicating age ≥65 years at sign-up, and (2) engagement with the app for at least seven days. |

Continued on next page

| Quantitative variables | 11 | Explain how quantitative variables were handled in the analyses. If applicable, describe which groupings were chosen and why | Materials and methods – Outcomes and data analysis | Analyses were conducted in R (RStudio version 2) and Microsoft Excel 2025 (Microsoft Corporation). Continuous variables (adherence, retention) were summarized using mean, standard deviation, median, and interquartile range. Categorical variables (feature usage) were summarized as counts and percentages. |
| --- | --- | --- | --- | --- |
| Statistical methods | 12 | (*a*) Describe all statistical methods, including those used to control for confounding | Materials and methods – Outcomes and data analysis | Analyses were conducted in R (RStudio version 2) and Microsoft Excel 2025 (Microsoft Corporation). Continuous variables (adherence, retention) were summarized using mean, standard deviation, median, and interquartile range. Categorical variables (feature usage) were summarized as counts and percentages. |
|  |  | (*b*) Describe any methods used to examine subgroups and interactions | n/a |  |
|  |  | (*c*) Explain how missing data were addressed | Materials and methods – Participant recruitment and data source | Key demographic variables such as date of birth and clinical conditions were optional. Users missing these fields were excluded from analyses requiring them. Analyses of medication adherence included only users who completed at least one medication task. For adherence calculations, days with no recorded adherence during active app use were treated as 0% until a user was inactive for three consecutive days, per our adherence definition. |
|  |  | (*d*) *Cohort study*—If applicable, explain how loss to follow-up was addressed  *Case-control study*—If applicable, explain how matching of cases and controls was addressed  *Cross-sectional study*—If applicable, describe analytical methods taking account of sampling strategy | n/a |  |
|  |  | (*e*) Describe any sensitivity analyses | Materials and methods – Participant recruitment and data source | A supplementary sensitivity analysis was conducted including all users with scheduled medication tasks regardless of engagement  duration to assess the impact of the ≥7-day inclusion criterion on adherence estimates. |
| Results | | | | |
| Participants | 13* | (a) Report numbers of individuals at each stage of study—eg numbers potentially eligible, examined for eligibility, confirmed eligible, included in the study, completing follow-up, and analysed | n/a |  |
|  |  | (b) Give reasons for non-participation at each stage | n/a |  |
|  |  | (c) Consider use of a flow diagram | n/a |  |
| Descriptive data | 14* | (a) Give characteristics of study participants (eg demographic, clinical, social) and information on exposures and potential confounders | Results - Participants | A total of 250 users were included in the analysis. The mean age was 70.1 years (SD = 4.7), with a range from 65 to 87, as seen in Table 1. Most participants were female (61.8%, n=155) and were located in Australia (61.6%, n=154). Users were enrolled across diverse sponsor programs, including health providers (36.8%, n=92), private insurers (20.8%, n=52), general health programs (20.4%, n=51), workers’ compensation (13.6%, n=34), and public health programs (8.4%, n=21). Among the 249 users who reported at least one clinical condition, most had multiple conditions, with 62 (24.9%) reporting one condition, 21 (8.4%) reporting two conditions, and 167 (67.0%) reporting three or more conditions, indicating a high prevalence of multi-morbidity in this cohort. Reported clinical conditions included heart and circulation conditions (17.9%, n=45), musculoskeletal and rheumatological conditions (11.8%, n=30), and other conditions such as allergies, obesity management, and chronic pain (15.8%, n=40). On average, users had 9.5 scheduled medication tasks, representing their prescribed medication regimen regardless of dosing frequency (e.g. daily, weekly, etc.) |
|  |  | (b) Indicate number of participants with missing data for each variable of interest | Results - Participants | Table 1 |
|  |  | (c) *Cohort study*—Summarise follow-up time (eg, average and total amount) | Results - Retention | Participants were retained in the app for a mean of over 2 years, 779.7 days (SD = 736.2), with a median of over one and a half years, 595.5 days (IQR = 206.3–1,182.8). The maximum retention observed was 2,465 days (Table 2). |
| Outcome data | 15* | *Cohort study*—Report numbers of outcome events or summary measures over time | Results |  |
|  |  | *Case-control study—*Report numbers in each exposure category, or summary measures of exposure | n/a |  |
|  |  | *Cross-sectional study—*Report numbers of outcome events or summary measures | n/a |  |
| Main results | 16 | (*a*) Give unadjusted estimates and, if applicable, confounder-adjusted estimates and their precision (eg, 95% confidence interval). Make clear which confounders were adjusted for and why they were included | Results |  |
|  |  | (*b*) Report category boundaries when continuous variables were categorized | Results |  |
|  |  | (*c*) If relevant, consider translating estimates of relative risk into absolute risk for a meaningful time period | n/a |  |

Continued on next page

| Other analyses | 17 | Report other analyses done—eg analyses of subgroups and interactions, and sensitivity analyses | Results – Medication adherence |  |
| --- | --- | --- | --- | --- |
| Discussion | | | | |
| Key results | 18 | Summarise key results with reference to study objectives | Discussion | In this multi-country, real-world cohort, older adults using the Perx app were observed to have high levels of engagement, retention, and adherence to their health tasks including medication adherence. Their usage patterns indicated relatively greater access to the social and leaderboard features of the app and less use of the rewards feature compared to Perx’s overall averages. |
| Limitations | 19 | Discuss limitations of the study, taking into account sources of potential bias or imprecision. Discuss both direction and magnitude of any potential bias | Discussion – Study strengths and limitations | This study also has some limitations. First, entering date of birth in the Perx app is optional, meaning some older users may have been not included in the study. Second, we were unable to establish baseline adherence rates prior to program use or to include a control group, given the retrospective design. Because participation in Perx is voluntary and analyses were restricted to users with at least seven days of engagement, selection and survivorship bias may be present, and observed adherence and retention estimates may be higher than would be expected in an unselected older adult population. Accordingly, findings should be interpreted as reflecting outcomes among users who engaged with the app. Additionally, feature engagement analyses were limited to counts of users accessing each feature in a given time period. While these provide insight into behavioral patterns, motivations for engagement were inferred rather than directly measured. Accordingly, findings are descriptive and associational, and causal inferences regarding the impact of specific app features cannot be made. |
| Interpretation | 20 | Give a cautious overall interpretation of results considering objectives, limitations, multiplicity of analyses, results from similar studies, and other relevant evidence | Discussion & Conclusion | In summary, older adults demonstrated high engagement, adherence, and retention in the Perx app, with sustained use far exceeding averages reported for digital health interventions more broadly. Their relatively higher use of social features suggests that digital programs aiming to support older adults should prioritize intrinsic and socially driven motivators, perhaps even more so than programs designed for other cohorts. These findings highlight the potential of mHealth solutions to empower older adults in managing their health and provide a foundation for future research exploring the specific design elements that best support this growing population. |
| Generalisability | 21 | Discuss the generalisability (external validity) of the study results | Discussion | Additionally, feature engagement analyses were limited to counts of users accessing each feature in a given time period. While these provide insight into behavioral patterns, motivations for engagement were inferred rather than directly measured. Accordingly, findings are descriptive and associational, and causal inferences regarding the impact of specific app features cannot be made. |
| Other information | |  | | |
| Funding | 22 | Give the source of funding and the role of the funders for the present study and, if applicable, for the original study on which the present article is based | Frontiers Statement | No Funding was received for the research and/or publication of this article |

*Give information separately for cases and controls in case-control studies and, if applicable, for exposed and unexposed groups in cohort and cross-sectional studies.

**Note:** An Explanation and Elaboration article discusses each checklist item and gives methodological background and published examples of transparent reporting. The STROBE checklist is best used in conjunction with this article (freely available on the Web sites of PLoS Medicine at http://www.plosmedicine.org/, Annals of Internal Medicine at http://www.annals.org/, and Epidemiology at http://www.epidem.com/). Information on the STROBE Initiative is available at www.strobe-statement.org.
