# Supplementary material for: Mobile health apps for older adults: real-world evidence on engagement and medication adherence
Source: Front Digit Health. 2026 Apr 17;8:1716880. doi: 10.3389/fdgth.2026.1716880 (PMC13132811; doi:10.3389/fdgth.2026.1716880)
Supplement: Supplementary file 2 [file Table1.docx]

**Supplementary Material**

**Table S1**

| **Analysis** | **N** | **Adherence Median (IQR)** | **Adherence Mean (SD)** |
| --- | --- | --- | --- |
| **Primary cohort** | 250 | 95.0 (85.3-98.3) | 86.2 (20.4) |
| **Sensitivity cohort (no ≥7 day rule)** | 267 | 94.4 (81.9-98.3) | 84.2 (22.3) |
